# Supplementary material for: BRCA2 binding through a cryptic repeated motif to HSF2BP oligomers does not impact meiotic recombination
Source: Nat Commun. 2021 Jul 29;12:4605. doi: 10.1038/s41467-021-24871-6 (PMC8322138; doi:10.1038/s41467-021-24871-6)
Supplement: Supplementary file 1 — Supplementary Information [file 41467_2021_24871_MOESM1_ESM.pdf]

SUPPLEMENTARY INFORMATION

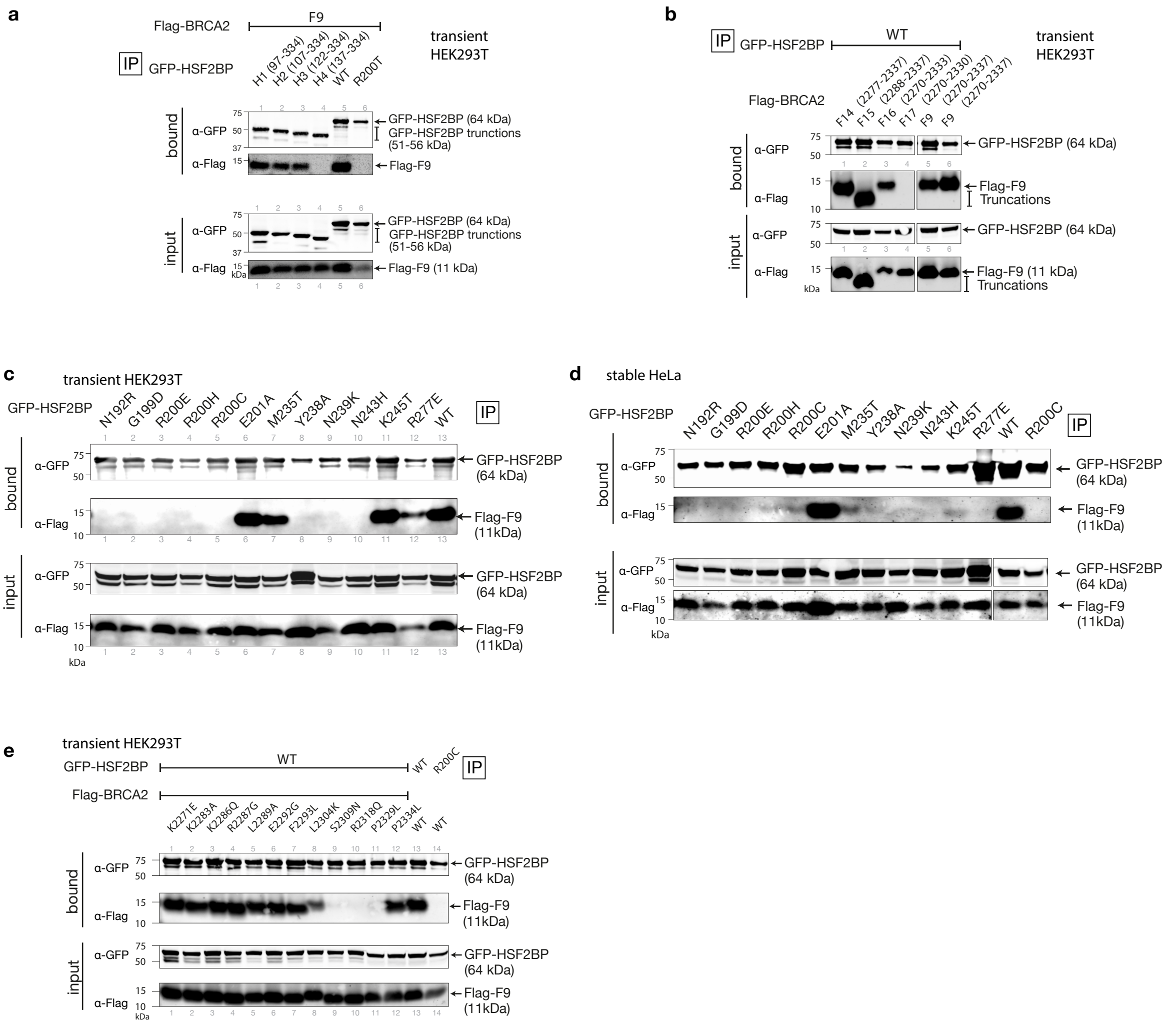

Supplementary Fig. 1 Co-immunoprecipitation of HSF2BP and BRCA2 variants a Full-length GFP-HSF2BP and its truncated versions (H1-H4) and Flag-tagged BRCA2 fragment F9 were transiently overproduced in HEK293T cells, co-immunoprecipitated using anti-GFP beads and analyzed by immunoblotting with anti-GFP and anti-Flag antibodies. **b** Full-length GFP-HSF2BP and Flag-tagged BRCA2 fragments were transiently overproduced in HEK293T cells, co-immunoprecipitated using anti-GFP beads and analyzed by immunoblotting with anti-GFP and anti-Flag antibodies. **c** Co-immunoprecipitation experiment from HEK293T cell lines transiently overproducing GFP-HSF2BP point mutants and a Flag-BRCA2 fragment corresponding to the previously identified minimal HSF2BP-binding domain (HBD, fragment F9, G2270-T2337)[16]. **d** As in panel (c), but fragments were produced by stable transformation of HeLa cells. **e** Co-immunoprecipitation experiment from HEK293T cell lines transiently overproducing GFP-HSF2BP and variants of the Flag-BRCA2-F9 fragment. Variant combinations shown in panels a-e were analyzed in at least two experiments with similar results.

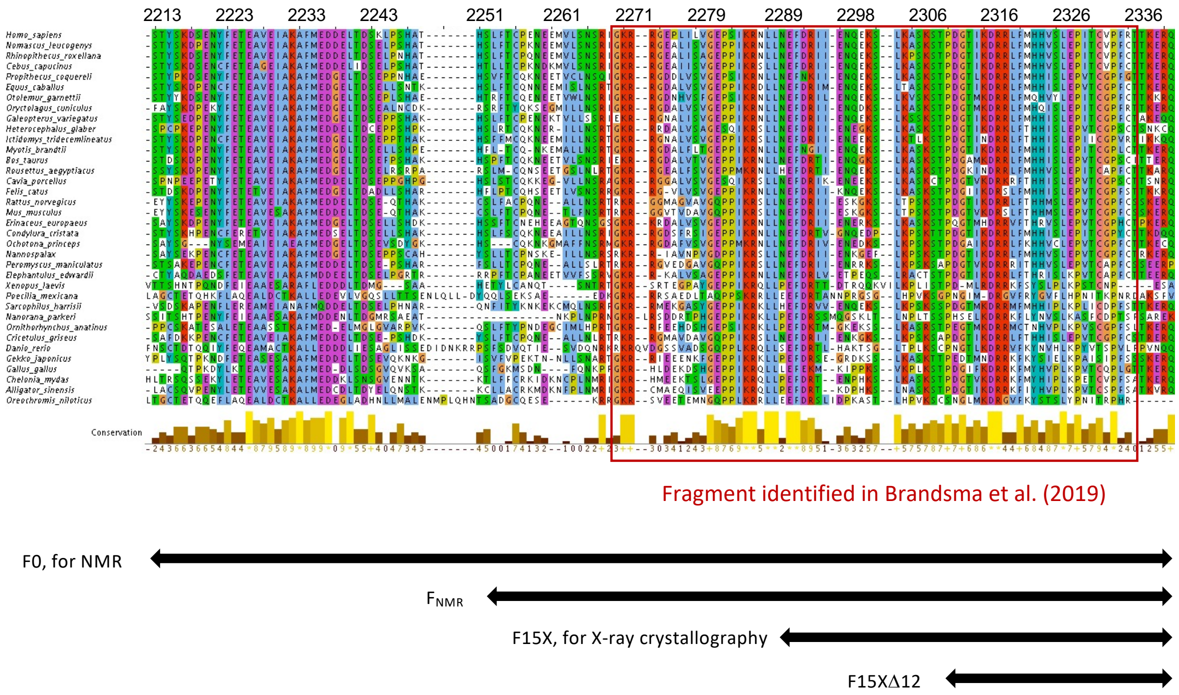

Supplementary Fig. 2 Sequence alignment of 36 BRCA2 homologs focused on region F0 (S2213-Q2342**) of human BRCA2.** Alignment and scoring were calculated using Jalview 2.10.1. The fragment previously identified as responsible for HSF2BP binding is boxed. The fragments produced for this study are indicated below.

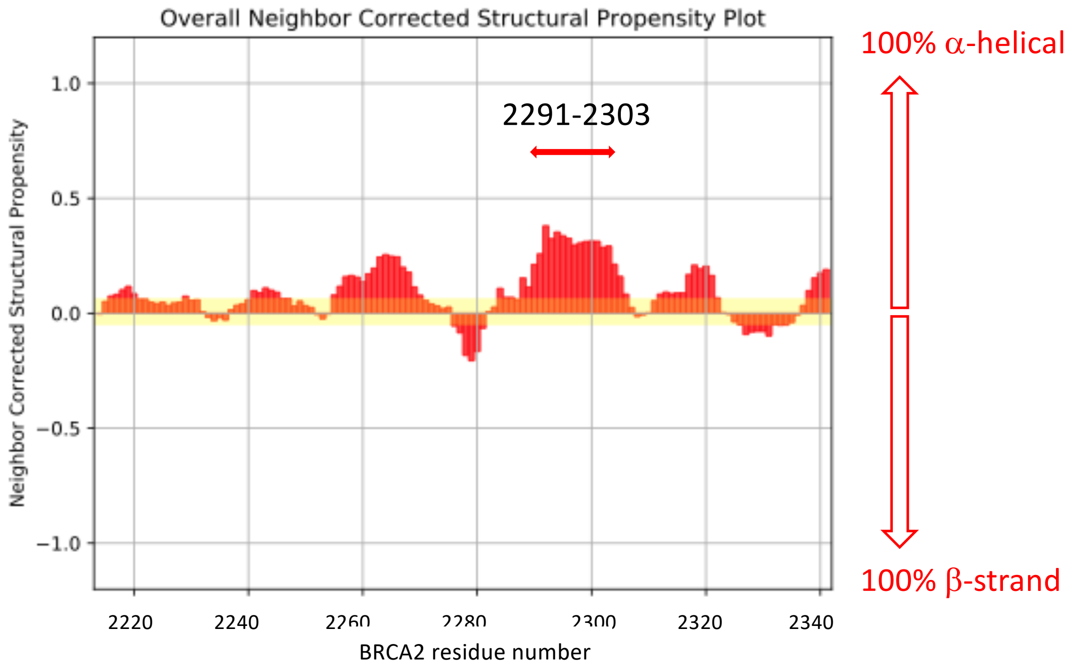

Supplementary Fig. 3 BRCA2 fragment F0 is disordered in solution. Analysis of the 3D heteronuclear NMR experiments recorded on the ^{15}N, ^{13}C labeled BRCA2 fragment F0 (S2213-Q2342**)** provided the chemical shifts for all Hn, N, Ca, Cb and Co nuclei. From these chemical shifts, the neighbor corrected structural propensity was calculated as a function of BRCA2 residue number, using the Webserver called "neighbor corrected structural propensity calculator" (https://st-protein02.chem.au.dk/ncSPC/; [64]). Positive and negative values correspond to populations with α-helical and β-strand conformations, respectively. In the case of BRCA2 F0, propensity values are all lower than 0.5, indicating that the protein is disordered in solution. Only region N2291-S2303 forms a transient α-helix present in more than 25% of the molecules.

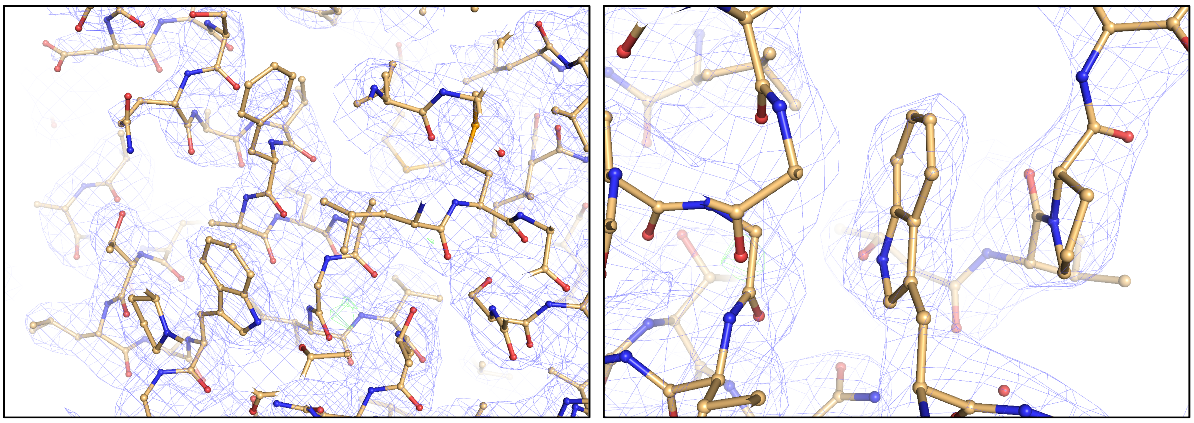

Supplementary Fig. 4 The crystal structure is nicely defined by the 2.6 Å resolution electron density. Example of the quality of the 2FoFc electron density map at 1.0 RMSD, showing a stacking between a tryptophane and a proline.

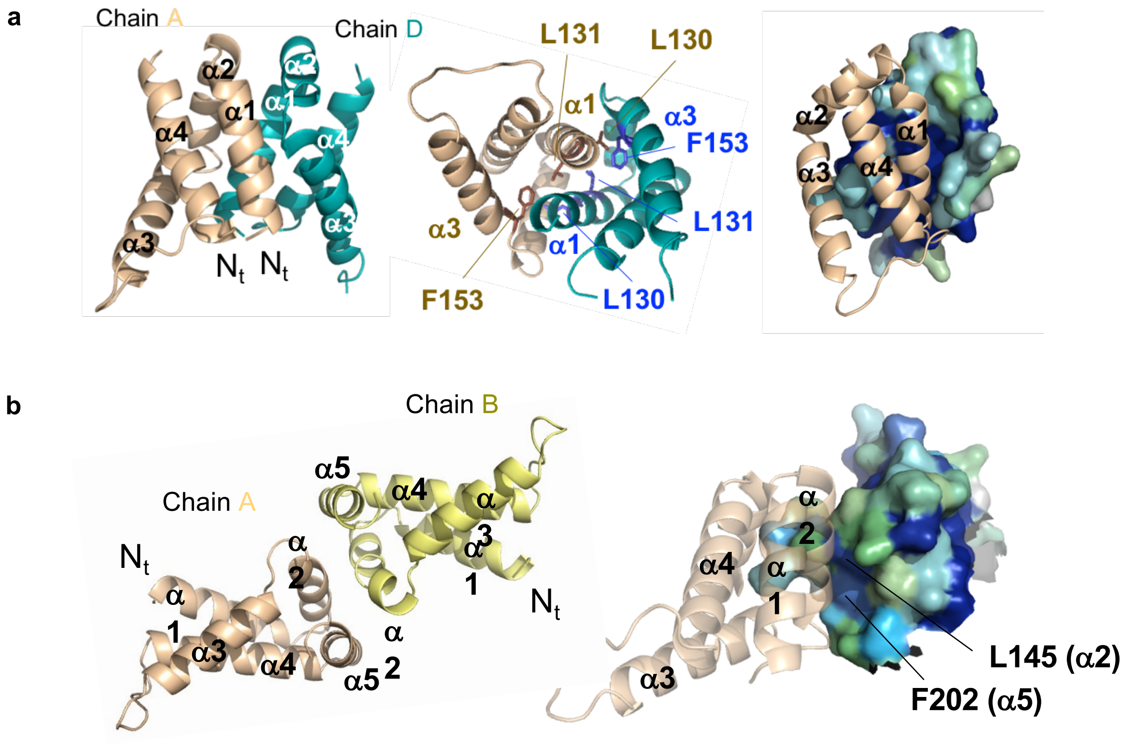

Supplementary Fig. 5 Oligomerization interfaces within the complex. a Dimerization interface observed between chains A and D. A similar interface is observed between chain B and C. In the left panel, the four N-terminal α-helices involved in the interface are displayed. In the middle panel, the three conserved residues of the interface are shown in sticks. In the right panel, the surface of chain D N-terminal helices is colored as a function of scores calculated by Consurf [62]. High, medium, weak and no conservation are indicated using dark blue, cyan, green and grey, respectively. **b** Central interface observed between chains A and B, resulting from the tetramerization of the ARM domains. Left panel: the symmetric interface of about 250 Å^2 involves helices α2 (K142, A143, K149 and A150) and helices α5 (N205, S206). Right panel: analysis of this interface using Consurf showed that it mostly comprises poorly conserved residues (see the surface representation of chain B). Only L145 in helix α2 and F202 in helix α5 are conserved; they interact with each other within each ARM chain, thus contributing to the interface between α2 and α5; however, they do not interact with conserved residues from the other chain.

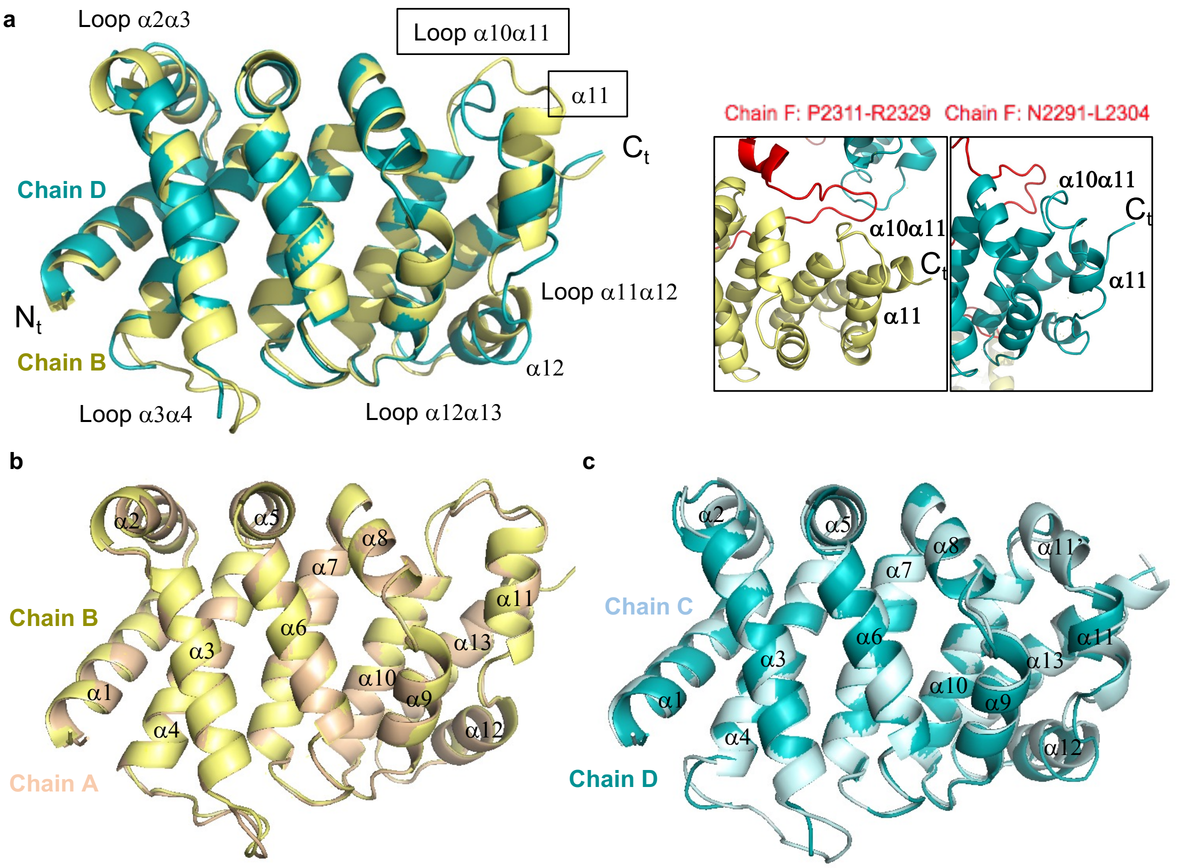

Supplementary Fig. 6 Structural variations are observed between ARM chains interacting with the N-terminal region of BRCA2 F15X and ARM chains interacting with the C-terminal region of BRCA2 F15X. a Superimposition of the structures of chains B and D, interacting with different parts of the BRCA2 peptide. Variations are observed, in particular in loop α10α11 and helix α11. These structural elements interact with either BRCA2 fragment P2311-R2319, or BRCA2 fragment N2291-L2304, as indicated in the boxed view. **b, c** Superimposition of the structures of (b) chains A and B, and (c) chains C and D, interacting with similar parts of the BRCA2 peptide. These structures are highly similar, as demonstrated by the low RMSD values measured between their backbone Cα atoms, displayed in Fig. 3a.

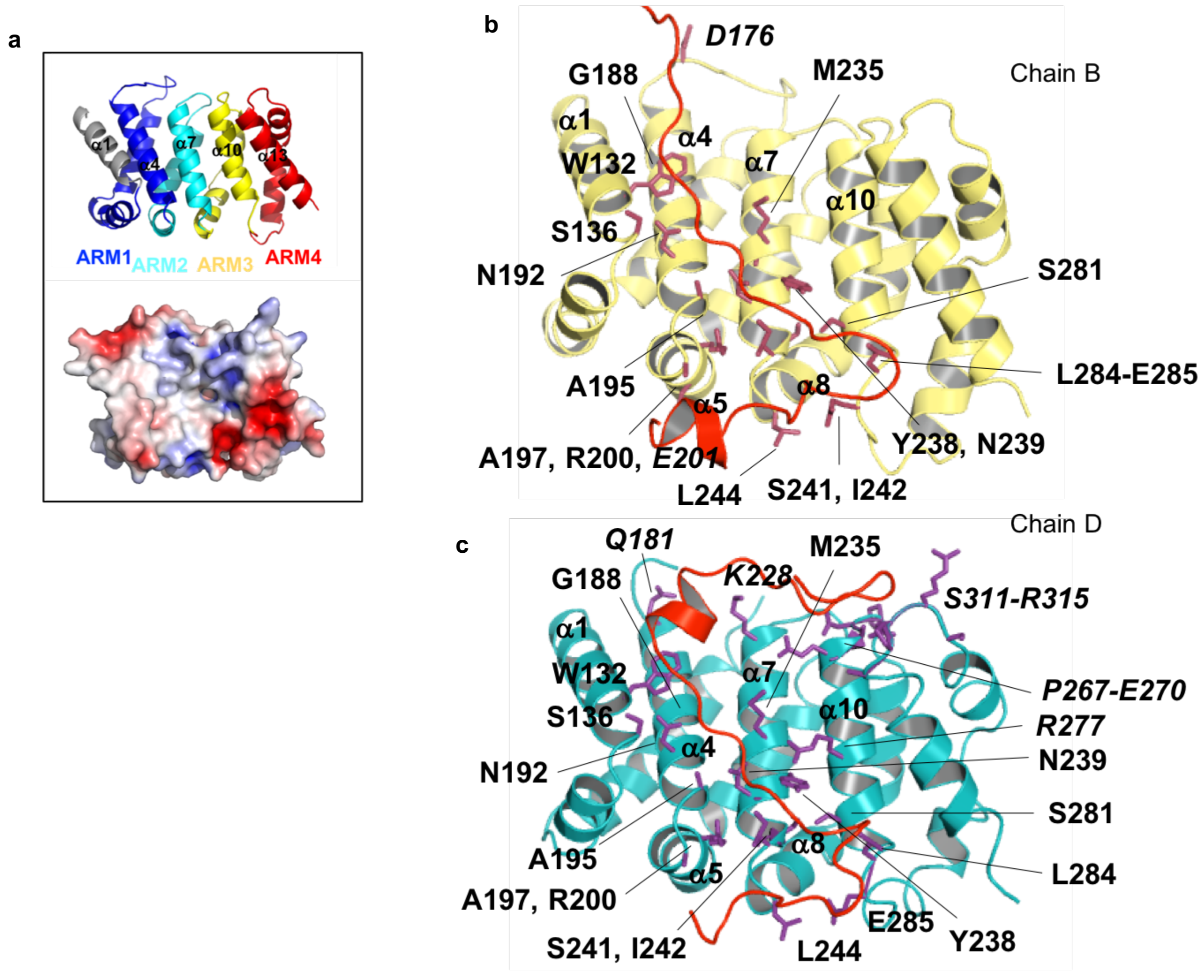

Supplementary Fig. 7 A set of ARM residues interact with both the N-terminal and C-terminal regions of the BRCA2 peptide. a Cartoon view of the four Armadillo repeats of ARM, colored in blue, cyan, yellow and red. The electrostatic potential at the surface of the domain is displayed at the bottom of the panel, to highlight the positively charged character of the groove defined by helices α1, α4, α7, α10 and α13. **b,c** Zoom on the interfaces between (b) chain B and the C-terminal region of F and (c) chain D and the N-terminal region of F. ARM residues that are either involved in hydrogen bonds or salt bridges with BRCA2, or buried by more than 30 Å^2 at the interface with BRCA2, are represented by colored sticks and labeled. Residues identified in only one of these two similar interfaces are labeled in italics.

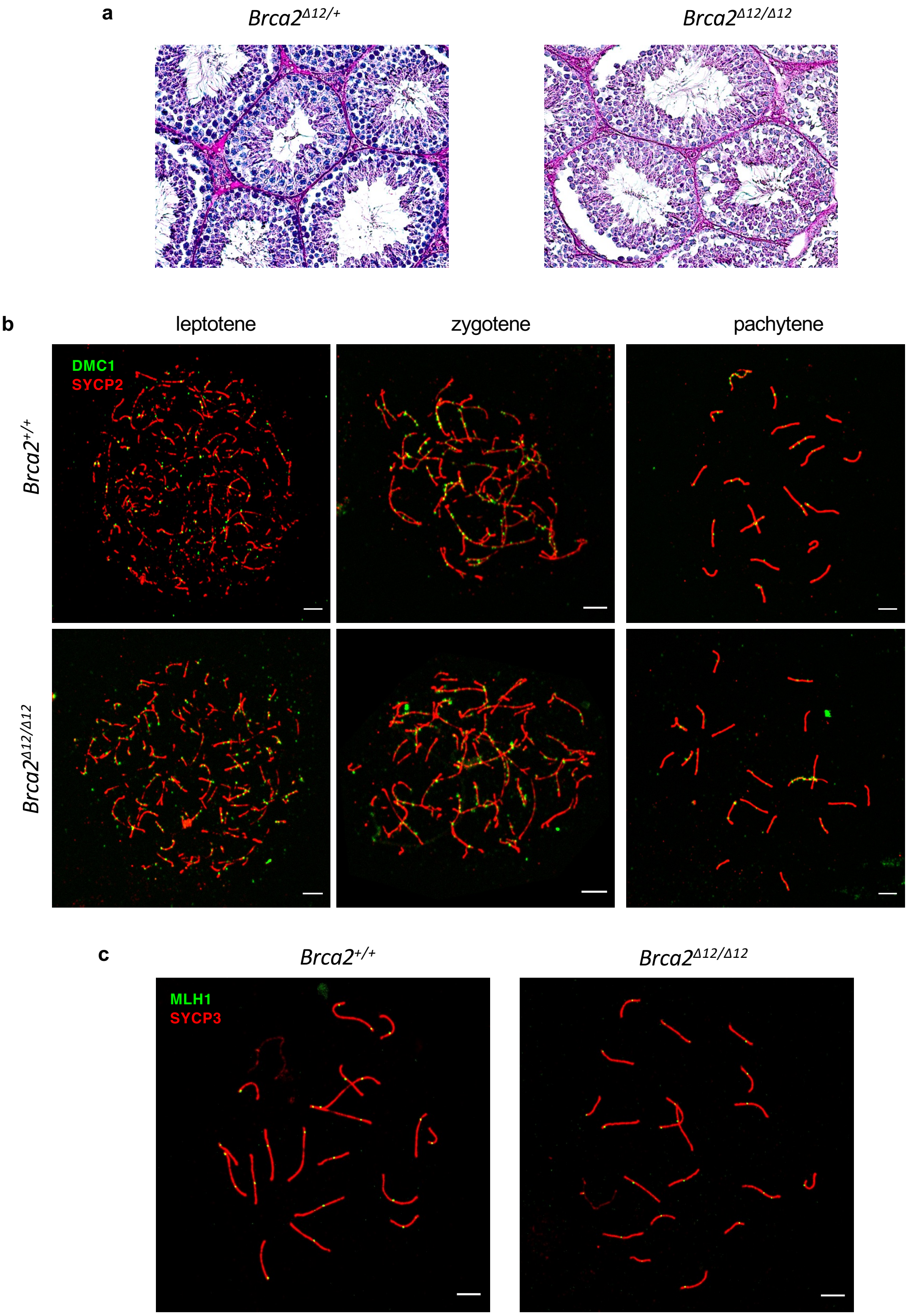

Supplementary Fig. 8 a Representative histological images of testis cross-sections. Two animals per genotype were analyzed in a single experiment. **b** Representative images used for DMC1 foci quantification in Fig. 6i,j. **c** Representative images used for MLH1 foci quantification in Fig 6k. Scale bars = 5 µm.

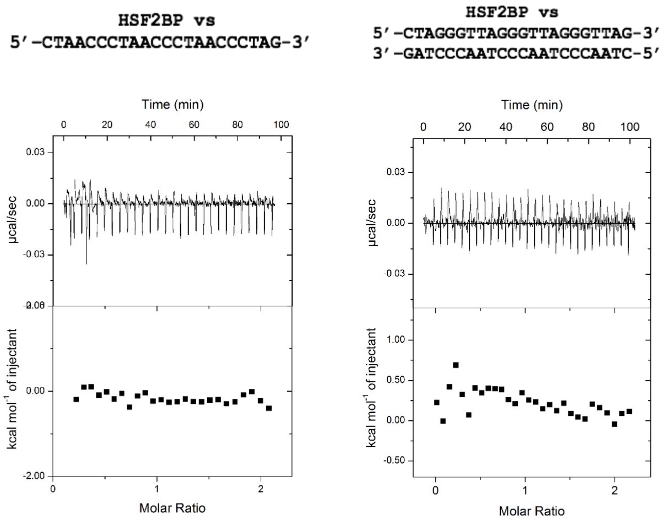

Supplementary Fig. 9 ITC analysis of the interactions between HSF2BP and DNA. HSF2BP (7-9 μM in the instrument cell) did not interact with ssDNA and dsDNA oligonucleotides (70-90 μM in the instrument syringe).

Supplementary Table 1. List of primers.

name	sequence 5'-3'
**mB2i11-F1**	AGCTGCCACATGGATTCTGAG
**mB2i12-R2**	GGACTAAGAGGCAAGGCATCA
**mB2e12-R1**	GCTTTTTGAAGGTGTTAAGGATTTT
**e11**	ACATTTTCTGATGTTCCTGT
**e12**	GTGCCATCTGGAGTGCTTTT
**e13**	GTCGTGAGCCGGTAAGATTG
**e14**	TCCCTGGAGACACTCAGCTT
**e15**	GAGCTGCTTAGGAGAACATGC
