# Supplementary material for: BRCA2 binding through a cryptic repeated motif to HSF2BP oligomers does not impact meiotic recombination
Source: Nat Commun. 2021 Jul 29;12:4605. doi: 10.1038/s41467-021-24871-6 (PMC8322138; doi:10.1038/s41467-021-24871-6)
Supplement: Supplementary file 6 — Description of additional supplementary files [file 41467_2021_24871_MOESM6_ESM.docx]

Description of additional supplementary information

Title: Supplementary Movie 1.

Description: Live cell recording of HSF2BP-GFP diffusion in the nuclei of Brca2+/+Hsf2bpGFP/GFP mouse ES cells.

Title: Supplementary Movie 2.

Description: Live cell recording of HSF2BP-GFP diffusion in the nuclei of Brca2∆12/∆12Hsf2bpGFP/GFP mouse ES cells.

Title: Supplementary Movie 3.

Description: Live cell recording of HSF2BP-GFP diffusion in the nuclei of Brca2∆12-14/∆12-14Hsf2bpGFP/GFP mouse ES cells.
