# Supplementary material for: BRCA2 binding through a cryptic repeated motif to HSF2BP oligomers does not impact meiotic recombination
Source: Nat Commun. 2021 Jul 29;12:4605. doi: 10.1038/s41467-021-24871-6 (PMC8322138; doi:10.1038/s41467-021-24871-6)
Supplement: Supplementary file 7 — Reporting Summary [file 41467_2021_24871_MOESM7_ESM.pdf]

nature research

Corresponding author(s): Alex N. Zelensky

Last updated by author(s): Jul 7, 2021

Reporting Summary

Nature Research wishes to improve the reproducibility of the work that we publish. This form provides structure for consistency and transparency in reporting. For further information on Nature Research policies, see our Editorial Policies and the Editorial Policy Checklist.

Statistics

For all statistical analyses, confirm that the following items are present in the figure legend, table legend, main text, or Methods section.

n/a	Confirmed	
☐	☒	The exact sample size (*n*) for each experimental group/condition, given as a discrete number and unit of measurement
☐	☒	A statement on whether measurements were taken from distinct samples or whether the same sample was measured repeatedly
☐	☒	The statistical test(s) used AND whether they are one- or two-sided<br>*Only common tests should be described solely by name; describe more complex techniques in the Methods section.*
☒	☐	A description of all covariates tested
☐	☒	A description of any assumptions or corrections, such as tests of normality and adjustment for multiple comparisons
☐	☒	A full description of the statistical parameters including central tendency (e.g. means) or other basic estimates (e.g. regression coefficient) AND variation (e.g. standard deviation) or associated estimates of uncertainty (e.g. confidence intervals)
☐	☒	For null hypothesis testing, the test statistic (e.g. *F*, *t*, *r*) with confidence intervals, effect sizes, degrees of freedom and *P* value noted<br>*Give P values as exact values whenever suitable.*
☒	☐	For Bayesian analysis, information on the choice of priors and Markov chain Monte Carlo settings
☒	☐	For hierarchical and complex designs, identification of the appropriate level for tests and full reporting of outcomes
☒	☐	Estimates of effect sizes (e.g. Cohen's *d*, Pearson's *r*), indicating how they were calculated

Our web collection on statistics for biologists contains articles on many of the points above.

Software and code

Policy information about availability of computer code

Data collection	Topspin 4.0, Origin 7.0
Data analysis	Graphpad Prism v9, ImageJ/FIJI v2.1, CellProfiler v4.1.3, CCPNMR 2.4, Origin 7.0 , XDS package , MOLREP (from CCP4), PHASER , BUCCANEER , BUSTER v5, PyMol v2.4

For manuscripts utilizing custom algorithms or software that are central to the research but not yet described in published literature, software must be made available to editors and reviewers. We strongly encourage code deposition in a community repository (e.g. GitHub). See the Nature Research guidelines for submitting code & software for further information.

Data

Policy information about availability of data

All manuscripts must include a data availability statement. This statement should provide the following information, where applicable:

- Accession codes, unique identifiers, or web links for publicly available datasets
- A list of figures that have associated raw data
- A description of any restrictions on data availability

The final pdb file and monoclinic data set have been deposited in the Protein Data Bank, entry code 7BDX. Source file for plots in Fig. 1,2,5,6 is provided. Microscopy images were deposited in BioStudies BioImage Archive under accession number S-BIAD166.

Field-specific reporting

Please select the one below that is the best fit for your research. If you are not sure, read the appropriate sections before making your selection.

☒ Life sciences ☐ Behavioural & social sciences ☐ Ecological, evolutionary & environmental sciences

For a reference copy of the document with all sections, see nature.com/documents/nr-reporting-summary-flat.pdf

Life sciences study design

All studies must disclose on these points even when the disclosure is negative.

Sample size	No statistical methods were used to predetermine sample sizes. We tested the hypothesis that Brca2 Δ12 should phenocopy Hsf2bp knock-out, so the number of imaged nuclei was based on the previous studies that revealed significant differences between Hsf2bp and wild-type strains (Zhang 2019 Nature Communications, Felipe-Medina 2020 eLife). Three animals were analyzed per genotype for the key comparison (+/+ vs Δ/Δ), based on the common practice in the field. Since this did not reveal significant differences, the number of Δ/+ was not increased after initial experiments and was kept at n=2. For per-nucleus analyses (Fig 6), nuclei were sampled equally from individual animals.
Data exclusions	No datapoints were excluded from quantitative comparisons.
Replication	Reported data represents at least two biological replicates: complete independent experiments performed on different dates, at least two independent animals were used per genotype, analyzed in at least two independent IF experiments, giving similar results.
Randomization	No randomization was performed. Assignment of experimental groups was based on genotypes. Littermate males were compared within experiments, so covariate control is not relevant.
Blinding	Quantitative image analysis was performed using using automated software scripts to avoid bias. In one case when this was not possible (Fig 6k), images were encoded and scored by three independent researches, as described in Methods. Blinding during image acquisition was not performed to ensure that sampling of meiotic prophase stages per animal per genotype was equal; slides were scanned in a systematic pattern. For other experiments (structure determination, biochemistry, immunoprecipitation) group assignment and blinding is not relevant.

Reporting for specific materials, systems and methods

We require information from authors about some types of materials, experimental systems and methods used in many studies. Here, indicate whether each material, system or method listed is relevant to your study. If you are not sure if a list item applies to your research, read the appropriate section before selecting a response.

Materials & experimental systems

n/a	Involved in the study	
☐	☒	Antibodies
☐	☒	Eukaryotic cell lines
☒	☐	Palaeontology and archaeology
☐	☒	Animals and other organisms
☒	☐	Human research participants
☒	☐	Clinical data
☒	☐	Dual use research of concern

Methods

n/a	Involved in the study	
☒	☐	ChIP-seq
☒	☐	Flow cytometry
☒	☐	MRI-based neuroimaging

Antibodies

Antibodies used	anti-GFP mAb (Roche, #11814460001, clones 7.1 and 13.1, 1:1000-5000), anti-GFP pAb (Abcam #ab290 and Invitrogen #A11122, 1:1000-1:5000), anti-RAD51 (Essers et al. 2002, 1:1000-1:10000), anti-BRCA2 mAb Ab1 OP-95 (Millipore #OP95, 1:1000), anti-BRCA2 (Abcam #27976, 1:1000), anti-Flag mAb (Sigma, F3165 and F1804, clone M2 1:1000-1:5000), anti-DMC1 (Abcam ab11054, clone 2H12/4, 1:1000), mouse anti-SYCP3 (Abcam ab97672, clone Cor 10G11/7, 1:5000), mouse anti-MLH1 (BD Pharmingen 51-1327GR, clone G168-15, 1:25), rabbit anti-HSF2BP (Felipe-Medina et al. 2020, antibody #1, 1:30) and rabbit anti- BRME1 (Felipe-Medina et al. 2020, antibody #2, 1:1000), rabbit polyclonal anti-SYCP3 (Lammers et al. 1994, 1:5000), guinea pig anti-SYCP2 (Winkel et al. 2009, 1:1000) guinea pig anti-HORMAD2 (Wojtasz et al. 2009, 1:100), sheep anti-BRCA2 (Min et al. 2012, 1:1000), anti-mouse CF680 (Sigma #SAB460199, 1:5000), anti-rabbit CF770 (Sigma #SAB460215, 1:5000), goat anti-guinea pig Alexa 546 (Invitrogen, A-11074 1:500), goat anti-rabbit Alexa 488 (Invitrogen, A-11008 1:500), goat anti-rabbit Alexa 546 (Invitrogen, A-11010 1:500), goat anti-mouse Alexa 488 (Invitrogen, A-11001 1:500), goat anti-mouse Alexa 555 (Invitrogen, A-21422 1:500), goat anti-mouse Alexa 633 (Invitrogen, A-21050 1:500), donkey anti-sheep HRP (ThermoFischer A16041, 1:5000).
Validation	All home-made antibodies (HSF2BP, BRME1) were validated using knock-out cell lines and/or mouse strains in previous publications (Brandsma 2019, Felipe-Medina 2020) and confirmed in this paper (Fig 6m-n). Other antibodies were validated and used in multiple previous publications.

Eukaryotic cell lines

Policy information about cell lines

Cell line source(s)	Parental mouse ES cell line was originally isolated de novo and described in a publication. HeLa and HEK293T cells used for protein production were the department stocks, originally obtained from ATCC.
Authentication	no authentication of the cell lines was performed other than PCR-genotyping for the indicated alleles.
Mycoplasma contamination	Mycoplasma tests are regularly performed in the cell culture facility; cell lines used in the study tested negative for mycoplasma infection.
Commonly misidentified lines (See ICLAC register)	No commonly misidentified cell lines were used in the study.

Animals and other organisms

Policy information about studies involving animals; ARRIVE guidelines recommended for reporting animal research

Laboratory animals	Laboratory mice (Mus musculus), C57BL/6 OlaHsd and derived knock-out strains, males and females. Mice were kept in IVC cages, under 12h dark/light cycle, with daylight from 7:00 – 19:00, ambient temperature is 19-24 ºC, 40-70% humidity.
Wild animals	No wild animals were used.
Field-collected samples	Field-collected samples were not used
Ethics oversight	All animals were kept in accordance with local regulations under the work protocols 17-867-11 and 15-247-20. Animal experiments were approved by the Dutch competent authority (Centrale Commissie Dierproeven, CCD) and all experiments conform to relevant regulatory standards.

Note that full information on the approval of the study protocol must also be provided in the manuscript.
