# Supplementary material for: BRCA2 binding through a cryptic repeated motif to HSF2BP oligomers does not impact meiotic recombination
Source: Nat Commun. 2021 Jul 29;12:4605. doi: 10.1038/s41467-021-24871-6 (PMC8322138; doi:10.1038/s41467-021-24871-6)

Figure 1c

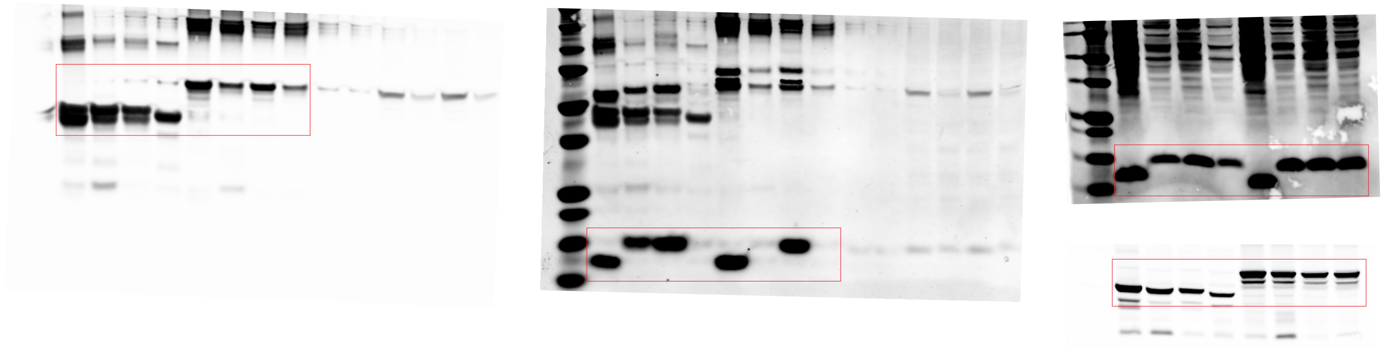

Figure 5d: medium resolution scan of the whole membrane

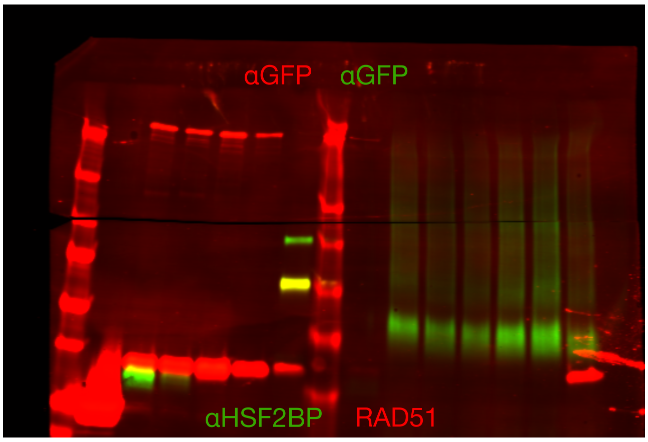

Figure 5d: high resolution rescan of ROIs:

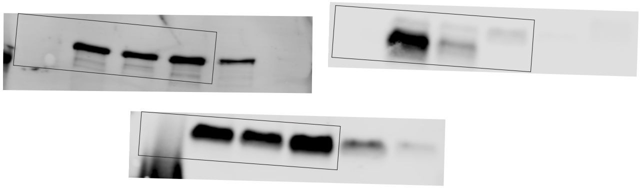

Figure 5e

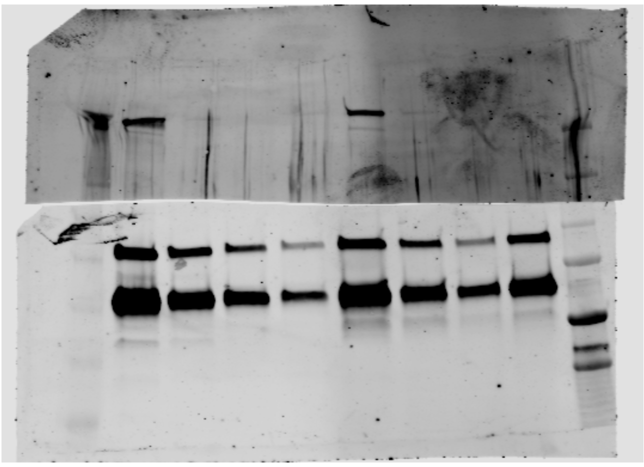

Figure 5f

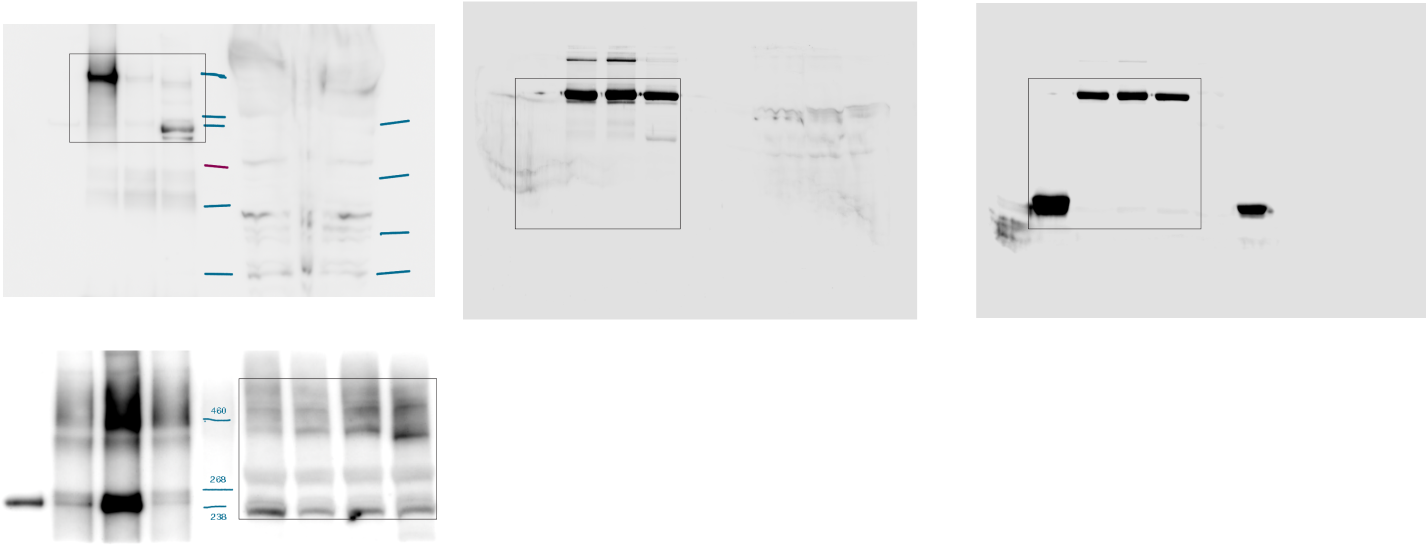

Figure 6c

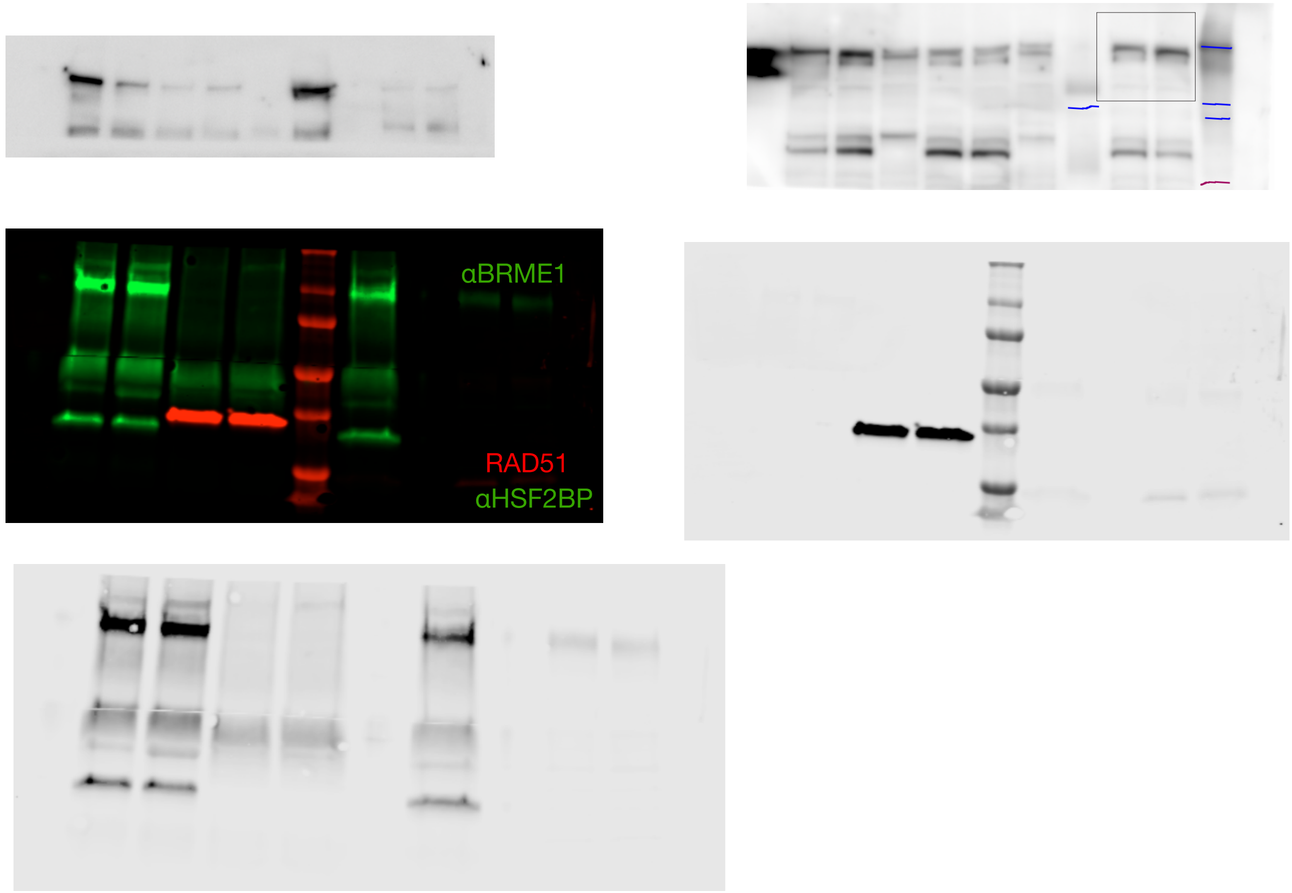

Figure S1a

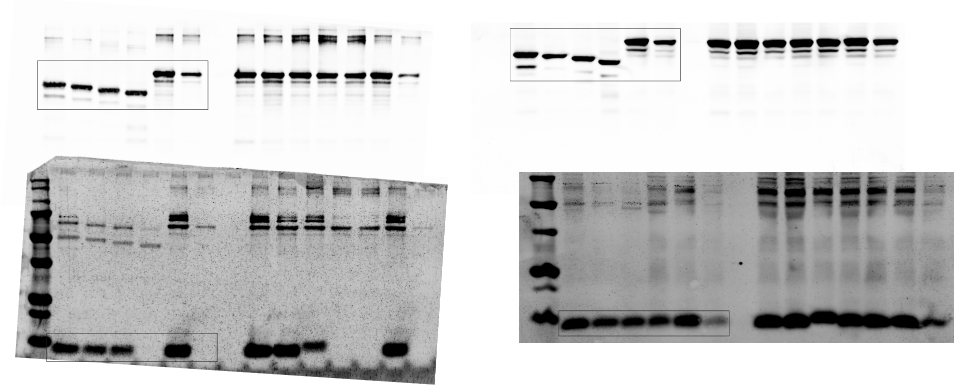

Figure S1c

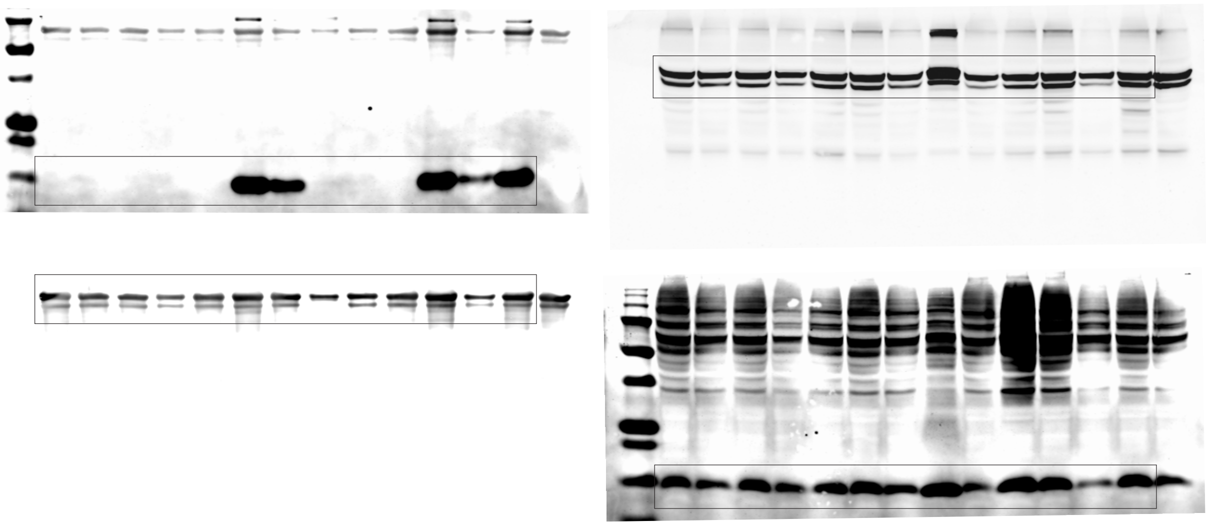

Figure S1b

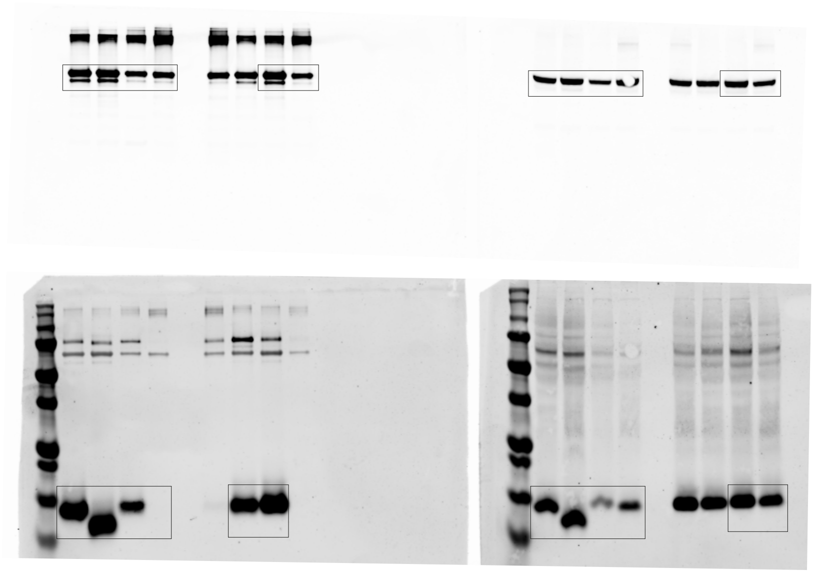

Figure S1d

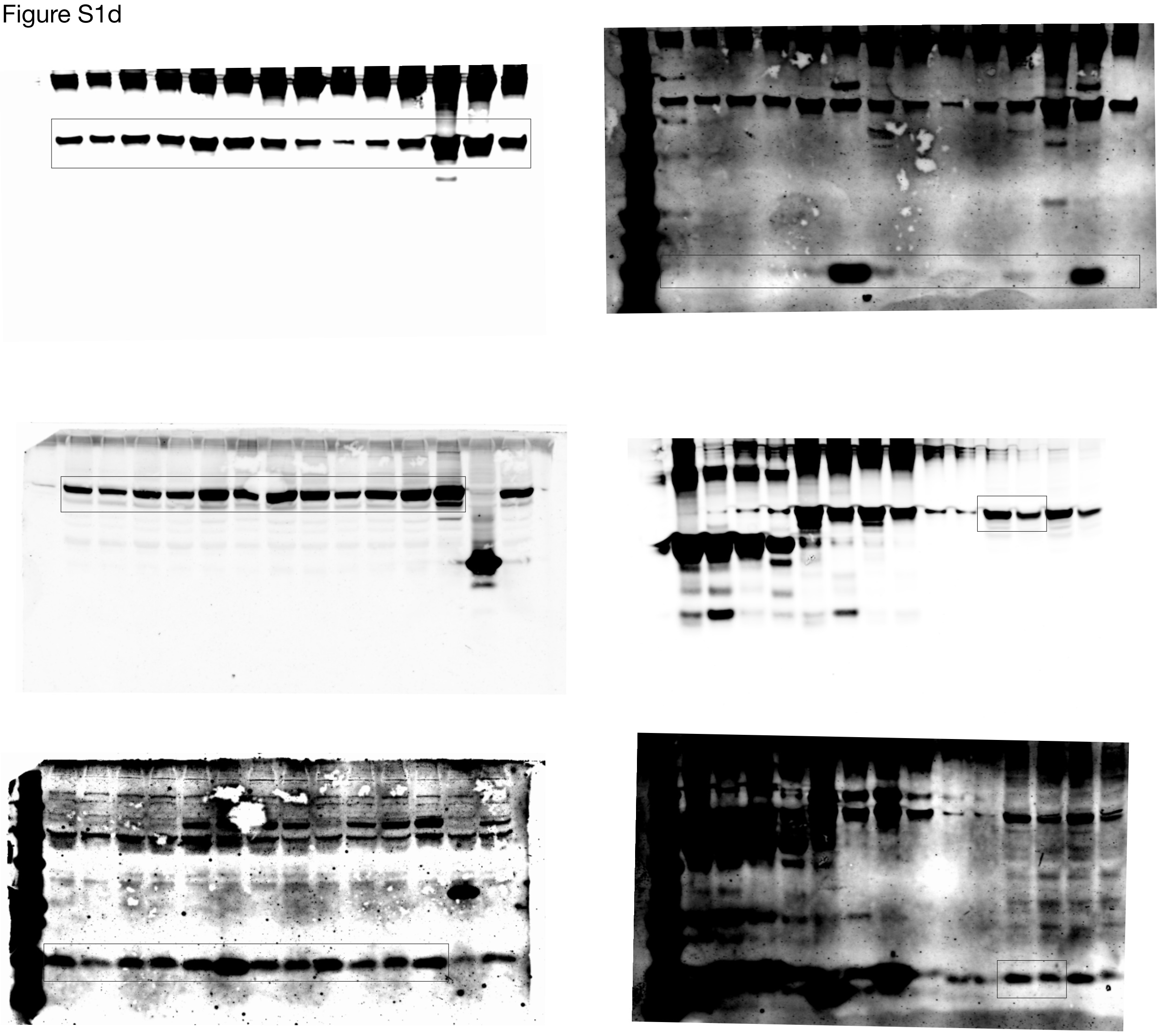

Figure S1e

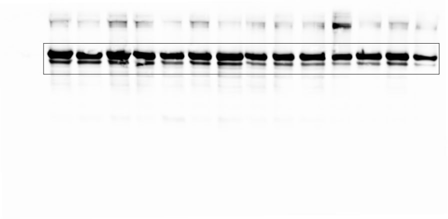

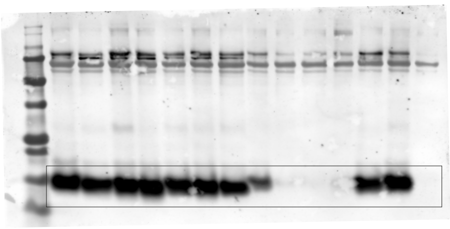

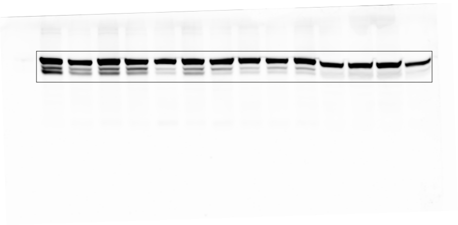

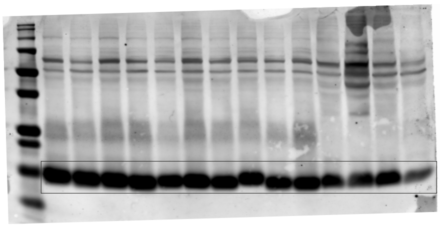

Supplement: Supplementary file 8 — Source Data [file 41467_2021_24871_MOESM8_ESM.zip › Uncropped blot images.pdf]
